# The impact of seasonal cattle grazing on ungulate spatiotemporal behavior in a multiuse recreational area in central Alberta

**DOI:** 10.1371/journal.pone.0313086

**Published:** 2024-11-01

**Authors:** Kathryn Knodel, Andrew Vanderleek, Lane Spyksma, Sierra Scheuermann, Darcy R. Visscher

**Affiliations:** 1 Department of Biology, The King’s University, Edmonton, Alberta, Canada; 2 Department of Biological Science, University of Alberta, Edmonton, Alberta, Canada; 3 Naturalis Biodiversity Center, Leiden, The Netherlands; Universidade Federal de Mato Grosso do Sul, BRAZIL

## Abstract

As grazing lands expand it is important to understand the effect cattle (*Bos taurus*) may have on native ungulates. Cattle presence in a landscape can cause both spatial and temporal partitioning in wild ungulates. We used remote cameras to investigate the impacts of seasonal rest-rotational cattle grazing on both the temporal and spatial behaviors of moose (*Alces alces*), elk (*Cervus canadensis*), mule deer (*Odocoileus hemonius*), and white-tailed deer (*Odocoileus virginianus*) in Cooking Lake-Blackfoot Provincial Park near Edmonton, AB, Canada. We found that all wild ungulates decrease their intensity of use in areas while cattle were grazing, and that this effect remains even after cattle have left, suggesting a degree of spatial partitioning. We also observed species specific changes in ungulate daily activity and nocturnality in response to cattle presence indicative of temporal partitioning. Elk increased their nocturnality while both deer species decreased their nocturnality. Understanding how cattle presence affects wild ungulates is essential for wildlife management, disease transmission, and conservation in the wake of potential increased ungulate-cattle interactions in the future.

## Introduction

Over a quarter of Earth’s land surface is utilized for livestock grazing and as grazing lands expand in response to increased demand for livestock products, wildlife and cattle will be found in closer proximity [1, 2]. Cattle grazing has the potential to drastically change landscapes, alter forage availability and impact wild ungulate behavior. These effects can be positive or negative depending on the habitat, forage availability, and wild game species involved [3, 4]. Proximity between wildlife and cattle can also have important consequences for zoonotic disease transmission [5–10]. Additionally, grazing lands are essential for conservation, offering open habitats that connect ecosystems, and allow for wildlife movement [1]. Thus, it is essential for wildlife management and conservation to better understand the factors that lead to co-existence between wild ungulates and cattle, and how foraging resources are partitioned spatially and temporally.

Cattle presence and grazing may result in dietary, spatial and temporal partitioning with wild ungulates. Competition for forage between ungulates and cattle may result in temporary spatial displacement through antagonistic behavioral interactions, which could cause spatial and/or temporal partitioning of foraging resources. These types of interactions have been shown to result in cattle temporarily displacing elk [11] and changes in deer diets when resources are limited [3]. Cooper et al. [4] found that white-tailed deer and cattle exhibited spatial overlap while still maintaining temporal partitioning allowing both able to utilize the most productive areas. However, this coexistence may require deer to modify their activity pattern in response to cattle grazing [12–14]. Cattle-ungulate interactions likely have one-sided impacts, as cattle seem to be indifferent to wild ungulates, whereas wild ungulates have a demonstrated preference for avoiding cattle [4, 15]. The spatiotemporal response of wild ungulates to cattle is likely mediated by dietary overlap with cattle. Although elk are generalists in terms of diet, graminoids are a large portion of their diet, so they have a significant overlap in diet with cattle [3]. Conversely, mule deer, white-tailed deer, and moose diets consist mainly of forbs and browse resulting in less competition with cattle [11].

Since the effects of cattle grazing on wild ungulates appears to be species-specific and context dependent, the ability to observe ungulate behavior in response to cattle presence at a single study site with multiple native ungulates provides a unique opportunity to understand spatial and temporal partitioning of wild ungulates in the face of seasonal cattle grazing. Therefore, in this study we used remote cameras in the Cooking Lake-Blackfoot Provincial Recreation Area in central Alberta, to study wild ungulate spatiotemporal responses to cattle grazed in a rest-rotation system in native pastures. Rest-rotation grazing systems involve mobilizing cattle to graze in different pastures for periods of time, while allowing various pastures a time period of ‘rest’ before cattle move into them [12]. We hypothesized that ungulates will 1) reduce their intensity of use of sites that are currently used by cattle (spatial partitioning), 2) modify their daily activity patterns in response to cattle presence (temporal partitioning), and 3) we expect a stronger spatiotemporal response from elk which have a greater dietary overlap with cattle.

## Materials & methods

### Study area

Our study was completed in the Cooking Lake-Blackfoot Provincial Recreation Area (hereafter, BPRA), a 97 km^2^ multiple use area about 40 km east of Edmonton, Alberta. A 2.2 m high perimeter fence surrounds the park limiting wildlife movement out of the park. Within the vegetated areas of the park are mixedwood forests, trails for recreational use, and various bodies of water. Vegetation is typical of the aspen parkland, consisting of stands of trembling aspen, *Populus tremuloides*, and open areas of native grassland [16]. The park is available for a variety of non-motorized activities including indigenous and licenced hunting, skiing, hiking, and biking. Cattle grazing occurs on approximately 4,000 hectares of grassland and is managed through a rough rest-rotational grazing system from May 15 until October 15 yearly. Cattle (stocked to a maximum of 5900 AUMs) are moved between fenced grazing pastures (mean (±SD) pasture size = 137.4 ± 45.3 hectares) within the BPRA as forage is depleted. Four species of ungulate are common in the park: elk, moose, white-tailed deer, and mule deer. Predators include occasional black bears (*Ursus americanus*), cougars (*Puma concolor*), grey wolves (*Canis lupus*), as well as the more common coyotes (*Canis latrans*).

### Camera setup

Remote cameras are an effective method for studying animal behavior efficiently in multispecies systems offering the ability to monitor the intensity of use at a site along with the temporal patterns in activity at a site [17]. As part of a larger project [18], 37 camera traps (Reconyx Hyperfire: H500, P800, P900) were deployed, serviced, and data was collected from them over a 13-month period from June 2017 to July 2018. Cameras were distanced at least 800m from each other and mounted on trees and fence posts to maximize wildlife and cattle detections. The cameras were serviced with new batteries and SD cards every 3 months. Whenever motion detection triggered the cameras, 3 consecutive images were captured. We used the EventFinder suite to filter non-target images and to collapse images into events, which were the unit of analysis [19]. In addition to the metadata collected by the camera itself, events were manually tagged to include species identification, demographic details, and count of individuals [18]. Research and collection permits for this non-invasive research were granted by Alberta Parks and the research was approved by The King’s University Research Ethics Board.

### Data analysis

For this analysis, we subset the data to only include cameras that were located within grazing pastures that held cattle for at least one week at some point during the grazing season (May 15-October 15). Events were compiled by ISO week as our measure of intensity of use for events containing cattle, mule deer, white-tailed deer, moose, and elk. The weeks when cattle were present were labelled as “during”. The “before” and “after” periods were each 3 weeks before and after the during grazing period, respectively. All statistical analysis was completed in R 4.2.1 [20].

#### Intensity of use

To assess the impacts of cattle presence on ungulate intensity of use of BPRA pastures (spatial partitioning), we compared intensities of use of moose, elk, mule deer, and white-tailed deer before, during, and after cattle presence. We created two generalized linear mixed models for the count data using a Poisson distribution [21] controlling for camera location as a random effect. In both models, elk functioned as the reference species, and “before” as the reference treatment. We used Akaike’s Information Criterion (AIC) to compare two models that included species and treatment period, with and without an interaction term. We assessed model fit using conditional R^2^ values from the ‘performance’ package [22].

#### Temporal activity patterns

We investigated the impacts of cattle grazing on the temporal activity patterns of ungulates (temporal partitioning), by comparing activity pattern and overlap of ungulates in each of the cattle grazing time periods (before, during, and after) using the ‘activity’ and ‘overlap’ packages [23, 24]. We compare the ungulate species activity overlap with cattle for each period keeping in mind that cattle are actually only present during the grazing period, but this gives us a baseline comparison to understand their shifts in activity pattern in a consistent fashion. The nocturnality, the average proportion of events of each ungulate capture during nighttime, was calculated for each species and treatment period [25]. Further, we calculated the risk ratio (RR) of changes in nocturnality between subsequent grazing periods (before to during (RR_bd_) and during to after (RR_da_)) where a risk ratio greater than one indicates increasing nocturnality and a value less than one, increasing diurnal activity [25].

## Results

Of the 37 cameras within the study area, 12 cameras met the criterion of recording an area in a grazing pasture and having cattle present for at least one week during the seasonal grazing period. From the 12 selected cameras, we observed 6536 cattle events, 407 elk events, 58 moose events, 294 white-tailed deer events, and 75 mule deer events.

**Fig 1:**
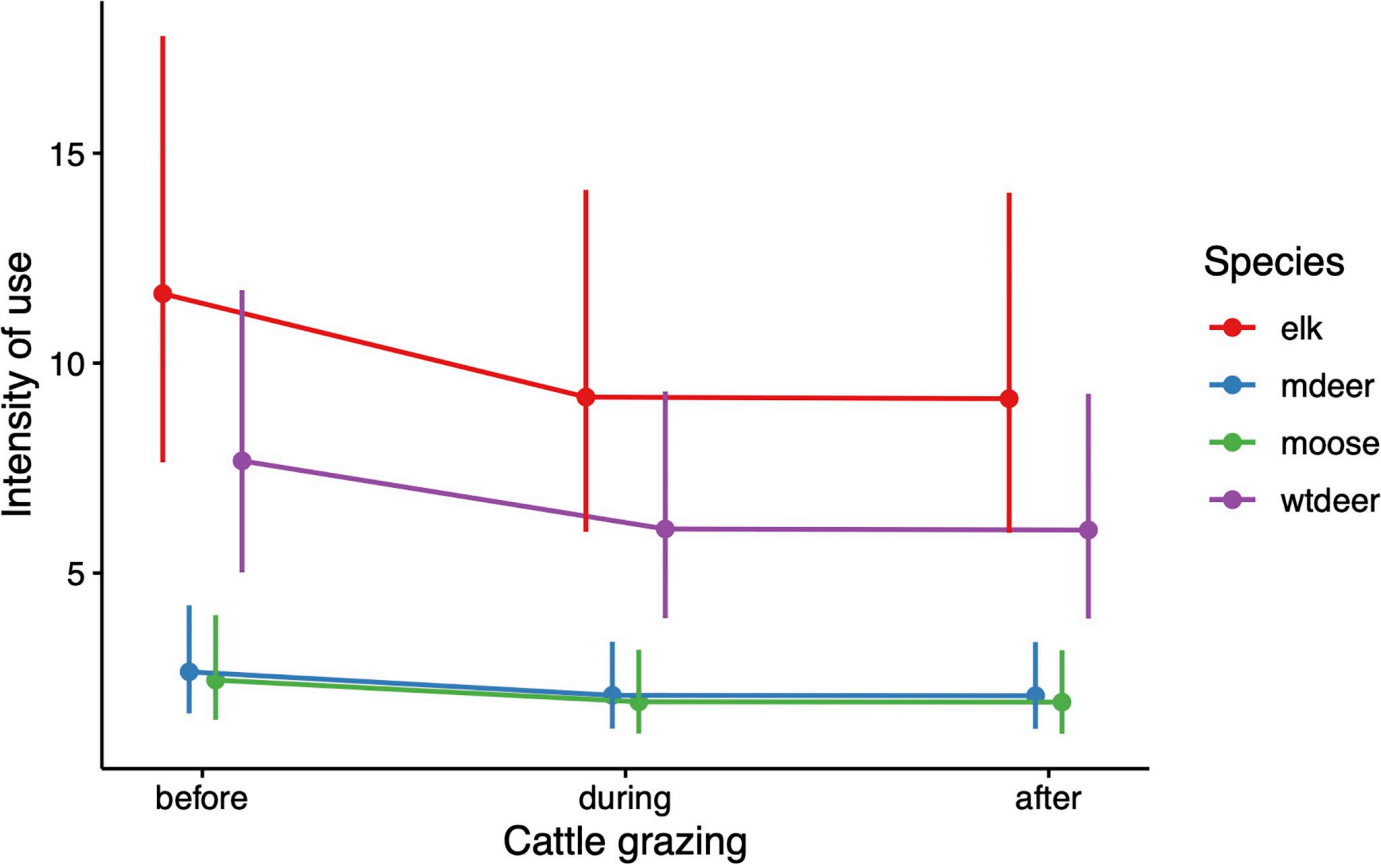
Impact of cattle grazing on ungulate intensity of use. Model predictions, and 95% confidence intervals of the weekly intensity of use by elk (pink), mule deer (green), and moose (blue), and white-tailed deer (purple) intensity of use by cattle grazing period, where “before” indicates the 3-week period before cattle grazing, “during” indicates the period in which cattle are present, and “after” indicates the 3-week period after cattle grazing.

We found that the model of intensity of use which did not include a species by grazing period interaction was more parsimonious than the model that did include the interaction (AIC = 693.79, *w*_*i*_ = 0.95, marginal R^2^ = 0.85) suggesting that all ungulates responded to cattle grazing in a similar fashion. Intensity of use differed for all ungulates, which reflects their general abundance and use of open areas in the park (all p<0.001 relative to elk). Ungulates showed a reduction in their intensity of use with the onset of cattle grazing (p = 0.005) which continued after cattle were moved out of the pasture (p = 0.004; Fig 1 and Table 1).

**Table 1:** General linear mixed model coefficients and significance for species-specific ungulate intensity of use as a function of cattle grazing period. Summary table of general linear mixed model describing the weekly intensity of use for each native ungulate species (where elk is the reference category and WT deer is white-tailed deer) and grazing period (where ‘before’ cattle grazing is the reference category), parameter estimates, their standard error (SE) and significance are given. Camera location was included as a random effect (variance = 0.488 and standard deviation = 0.699), the model had strong support (wi = 0.95) and fit the data well (AIC = 693.790, logLik = -330.890, Conditional R^2^ = 0.40, and Marginal R^2^ = 0.86).

Variable	Estimate	SE	p-value
Intercept	2.456	0.216	< 0.001
Mule deer	-1.481	0.126	< 0.001
Moose	-1.559	0.142	< 0.001
WT deer	-0.418	0.077	< 0.001
During	-0.238	0.084	0.005
After	-0.242	0.084	0.004

**Fig 2:**
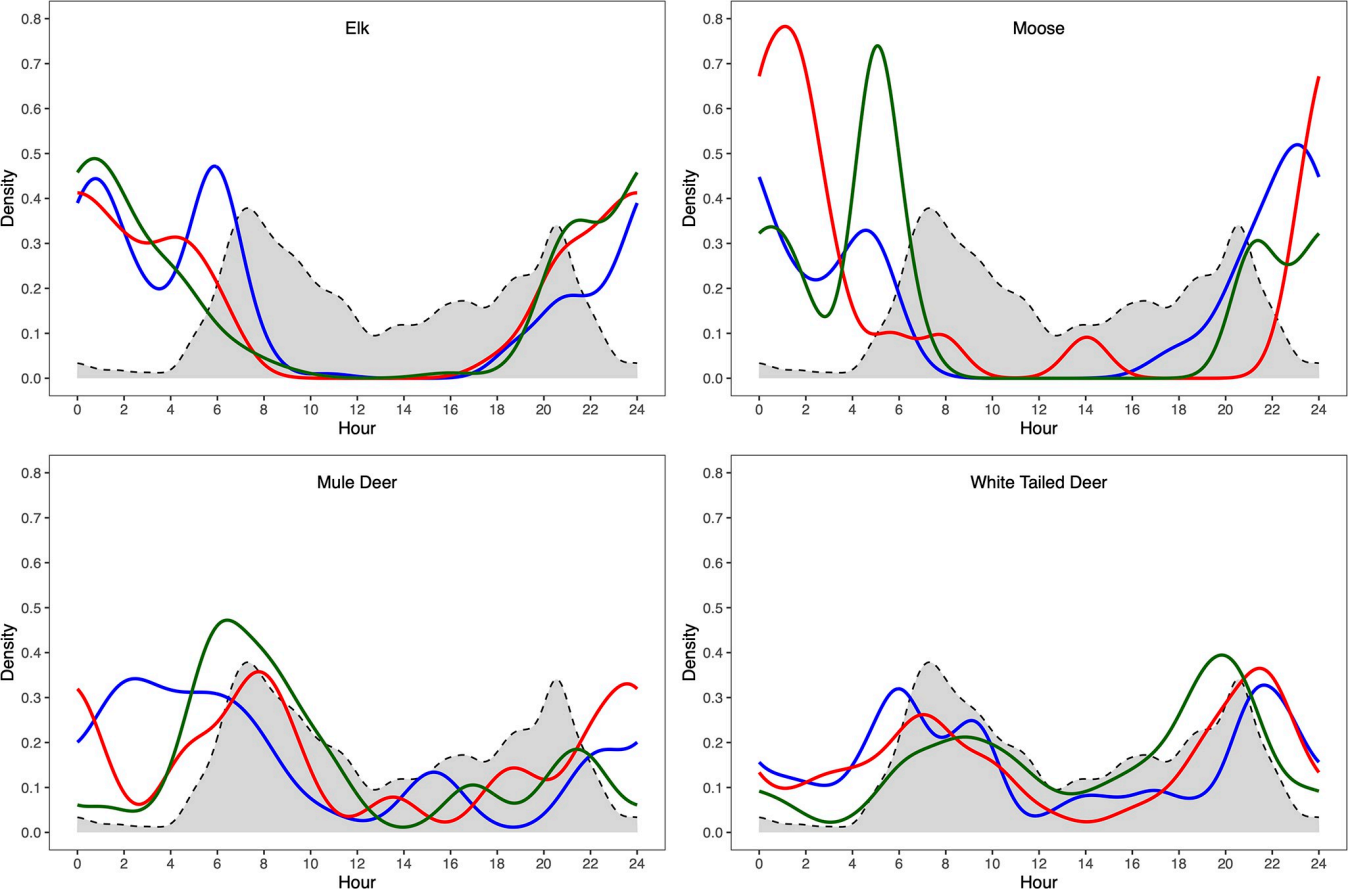
Patterns of daily activity of native ungulate relative cattle activity and grazing period. Daily temporal activity patterns for each of the study species, in which the blue lines represent daily activity before cattle grazing, red lines indicate daily activity during cattle presence, green line indicates daily activity after cattle grazing, and the dashed line and filled region indicates cattle daily activity.

The temporal partitioning response of ungulates to cattle grazing was species specific (Fig 2). Before cattle presence, elk, mule deer, white-tailed deer, and moose showed an average nocturnality of 54.8%, 55.9%, 33.3% and 77.3%, respectively. Elk activity overlap with cattle decreased with the onset of grazing (before = 0.404 and during = 0.369) and continued to decline following the removal of cattle (after = 0.346). This was due to an increase in nocturnal behaviour (RR_bd_ = 1.287) that continued beyond the removal of cattle (RR_da_ = 1.035). Moose activity overlap with cattle decreased with the onset of grazing (before = 0.346 and during = 0.232) but started to rebound following the removal of cattle (after = 0.313). This was due to an initial slight increase in nocturnal behaviour (RR_bd_ = 1.078) with the presence of cattle, which once removed resulted in a slight shift to more diurnal activity (RR_da_ = 0.933). White-tailed deer increased their overlap with cattle through each period (before = 0.683, during = 0.721, and after = 0.840), the result of increasing their diurnal activity between each period (RR_bd_ = 0.805 and RR_da_ = 0.446). Mule deer followed a similar trend to white-tailed deer increasing their overlap with cattle through each period (before = 0.516, during = 0.671, and after = 0.726), again, the result of increasing their diurnal activity between each period (RR_bd_ = 0.737 and RR_da_ = 0.405).

## Discussion

In the fully fenced and multiuse BPRA, we showed that wild ungulates including white-tailed deer, mule deer, moose, and elk all decreased their intensity of use in pastures when cattle were present and grazing. We found that this effect continued in the three-week period after cattle left and did not rebound to the intensity of use recorded at those sites prior to cattle grazing. Our results are consistent with previous studies increased home range sizes during cattle grazing periods, and avoidance or reduced use of areas in which cattle were present [15, 26–28]. As we expected, elk were more affected by cattle presence than the other three species, likely because elk diet overlap more with cattle than the other ungulates. In previous studies elk have been observed to spatial partitioning from cattle by selecting areas where cattle were absent, in contrast with mule and white-tailed deer who were less impacted by cattle presence [12, 29, 30]. We found that cattle grazing had lingering effect on ungulate use, however this may be in part due to the forage depletion that also accompanies cattle presence or that the rotational nature of the grazing does not give sufficient time for ungulates to acclimatize to cattle presence. Indeed, white-tailed deer have been shown to share pastures with cattle in continuous grazing systems while avoiding cattle in shorter term grazing systems [4].

While studies have noted the importance of temporal partitioning for ungulates in response to cattle grazing, typically with increase in nocturnal behaviour, our results suggest diverging pattern of temporal partitioning among wild ungulates [4, 28]. We found that, consistent with the literature, elk and moose become more nocturnal decreasing their activity overlap with cattle, however both mule and white-tailed deer become more diurnal and increase their activity overlap with cattle. As hypothesized, we found stronger partitioning in elk whose diet more strongly overlaps with cattle [3]. Beyond affecting daily activity patterns in ungulates cattle may also change the amount of time spent feeding and alert, together these changes may have consequences for thermoregulation, predation, and foraging efficiency [13, 14, 28]. Indeed, the temporal decisions that mule and white-tailed deer may weigh the trade-offs of avoiding predators such as coyotes by avoiding nocturnal activities in open areas or by associating with cattle in addition to avoid human recreation in this multiuse landscape [18, 28]. Cattle grazing may contribute to increased competition and social interactions between wild ungulates. Elk are known to have antagonistic interactions with deer resulting in deer avoiding locations with high elk density [31]. It maybe that the increased overlap with cattle through diurnal activity of deer in our study area is a response to elk avoidance more than cattle presence, however we are unable to rule out a faciliatory foraging relationship with cattle [32]. Cattle management in the BPRA includes the presence of range riders to protect cattle from the potential of depredation. Previous research in the BPRA has found that the presence of humans has more of an impact on wild ungulates compared to predator presence [18] and it may be difficult to untangle the effects of cattle grazing from human presence associated with the cattle or other recreation activities as well as predation.

Beyond spatiotemporal displacement and competition for foraging resources, our results provide insight into the potential for disease transmission which may occur between wildlife and livestock, which is of increasing global concern due to urbanization [5–7]. Recent work in Alberta focused on elk-cattle interactions suggests that indirectly transmitted pathogens co-occurred in elk and cattle, while species that rely on direct transmission were uninfluenced by overlap between cattle and elk [8]. In our system there was much more opportunity and evidence of overlap at small spatial and temporal scales (ungulates in the same field at the same time as cattle), suggesting the direct transmission could be a concern. We suggest that care should be taken to introduce further attractants that may promote overlap [9, 10].

## Conclusions

We found that cattle presence in rest-rotationally grazed pastures in Cooking Lake-Blackfoot Provincial Park resulted in decreased intensity of use and changes in activity patterns for all four ungulate species found in the park. The spatiotemporal behavior we observed suggests that competition for resources and social interactions may constrain ungulate behavior. Notably we found that these effects continued even after cattle grazing at a site had ceased suggesting that grazing effects extend beyond simple cattle presence. The management implication of this change in spatiotemporal behavior may be particularly problematic in a small fenced multiuse park where ungulates are already constrained by the perimeter fence and high levels of recreational activities, including hunting as well as predation. As cattle grazing is anticipated to increase in future and put larger strain on ungulate species, further research to better understand these impacts, including disease transmission, will be essential to mitigate their effects. This may require new rangeland management strategies, potentially including increased rest periods for grassland areas, and minimized cattle grazing where possible.
